# Facile Preparation of Highly Stretchable TPU/Ag Nanowire Strain Sensor with Spring-Like Configuration

**DOI:** 10.3390/polym12020339

**Published:** 2020-02-05

**Authors:** Wei Pan, Juan Wang, Yong-Ping Li, Xiao-Bo Sun, Jin-Ping Wang, Xiao-Xiong Wang, Jun Zhang, Hai-Dong You, Gui-Feng Yu, Yun-Ze Long

**Affiliations:** 1 College of Chemistry and Pharmaceutical Sciences, Qingdao Agricultural University, Qingdao 266109, China; tcpanwei@126.com (W.P.); xbsun@qau.edu.cn (X.-B.S.); jpwang@qau.edu.cn (J.-P.W.); 2 College of Science and Information, Qingdao Agricultural University, Qingdao 266109, China; wangjuan7712@126.com (J.W.); yongpli@126.com (Y.-P.L.); hdyou@qau.edu.cn (H.-D.Y.); 3 Collaborative Innovation Center for Nanomaterials & Devices, College of Physics, Qingdao University, Qingdao 266071, China; wangxiaoxiong69@163.com (X.-X.W.); iamzhangjun@163.com (J.Z.); 4 State Key Laboratory of Bio-Fibers and Eco-Textiles, Qingdao University, Qingdao 266071, China

**Keywords:** spring-like configuration, electrospinning, stretchable, sensor

## Abstract

Stretchable nano-fibers have attracted dramatic attention for the utility in wearable and flexible electronics. In the present case, Ag nanowires (AgNWs)-intertwined thermoplastic polyurethanes (TPU) unwoven nano-membrane is fabricated by an electrospinning method and dip coating technique. Then a strain sensor with a spring-like configuration is fabricated by a twisted method. The sensor exhibits superior electrical conductivity up to 3990 S·cm^−1^ due to the high weight percentage of the Ag nanowires. Additionally, thanks to the free-standing spring-like configuration that consists of uniform neat loops, the strain sensor can detect a superior strain up to 900% at the point the sensor ruptures. On the other hand, the configuration can mostly protect the AgNWs from falling off. Furthermore, major human motion detection, like movement of a human forefinger, and minor human motion detection, such as a wrist pulse, show the possible application of the sensor in the field of flexible electronics.

## 1. Introduction

Flexible wearable devices have gained considerable attention and experienced rapid growth in recent years [1,2,3,4,5,6,7,8]. Many efforts have been dedicated to integrate the attributes of low cost, lightweight, stability, small size, flexibility, and multi-functional sensitivity to the flexible sensor, which is an important branch of wearable devices [4,5,6,7,8,9]. However, high conductivity and high mechanical stretchability are hard to attain simultaneously [10].

Up to today, two strategies have been adopted to increase the stretchability of the devices [7,9,11]. One is exploring and adopting intrinsically flexible or stretchable materials. For example, Wujcik et al. fabricated a piezoresistive strain sensor based on a poly(2-acrylamido-2-methyl-1-propanesulfonic acid) (PAAMPSA)/polyaniline (PANI)/phytic acid (PA) polymer complex. The strain sensor owned the characteristics of self-healing without external stimuli and a high stretchability of 1935% [8]. Additionally, a stretchable substrate, such as polydimethylsiloxane (PDMS), is introduced to overcome the limitations to some extent, e.g., the stretchability of a semi-embedded wrinkled Ag nanowires (AgNWs)/PDMS-network conductive film was 300% [12], and an array of capacitive sensors based on the stretchable silver nanowire and PDMS can detect a strain up to 50% [13]. However, the stretchability of the sensor is limited by the mechanical fracture limit of PDMS [14]. The other is carrying out novel configuration designs, including percolation network, island bridge, serpentine configuration, sponge structure, chess-like configuration, twisted configuration, and buckled configuration [11,15,16,17,18,19,20,21,22,23,24]. For example, cracked cellulose nanofibril/silver nanowire layer-coated polyurethane (PU) sponge was acquired through a simple dip-coating process, and the obtained strain sensor exhibited a high sensitivity over a detection range of 50% compression strain due to the high porosity and good elasticity of the polymer matrix [2]. Compared to other configurations, the wavy, buckled, and wrinkled configurations are relatively simple to attain [9], and the chess-like configuration is relatively popular. However, the stretchability of sensors with the above configurations is relatively low compared to that of those with the spring-like configuration [7,25].

Graphene, carbon nanotubes, conducting polymer, metal nanowires, and metal nanomaterials have shown to be promising building blocks to increase the conductivity of the devices extensively [26,27,28]. In particular, a silver nanowire, which is a unique conductive nanomaterial with high conductivity and feasibility of large-scale production, has been studied as one of the most promising flexible conducting materials for flexible strain sensors [28,29,30]. However, several challenging issues also remain. For example, wearable electronics must deal with complicated situations with various deformations, which can cause AgNWs to fall off, and this will greatly hinder the stability of the electronic device. Therefore, the poor adhesion between the AgNWs and the flexible substrates is an important limitation that we must face [31,32]. Over-coating layer on the AgNWs, peel-assembly-transfer strategy, etc., have been adopted to solve the problem. Nevertheless, the procedure is costly and the performance of the flexible electronic devices can be affected [32].

In this work, we designed a strain sensor with high stretchability and unique conductivity. A AgNWs/thermoplastic polyurethanes (TPU) nano-membrane was fabricated by a facile electrospinning technique and dip coating method, and then a sensor with a spring-like configuration was obtained through the twisted method. Taking advantage of the spring-like configuration and the intertwine of the AgNWs, the maximum elongation at break and highest conductivity of the sensor were 900% and 3990 S·cm^−1^, respectively. In the procedure of fabricating the spring-like configuration, AgNWs can mostly be rolled into the hierarchically arranged fibers to enhance the stability between the substrate and the conducting AgNWs. Additionally, the unique achievements of detecting human motion implied the great application of the new spring-like configuration in stretchable sensors.

## 2. Experimental Section

**Figure 1:**
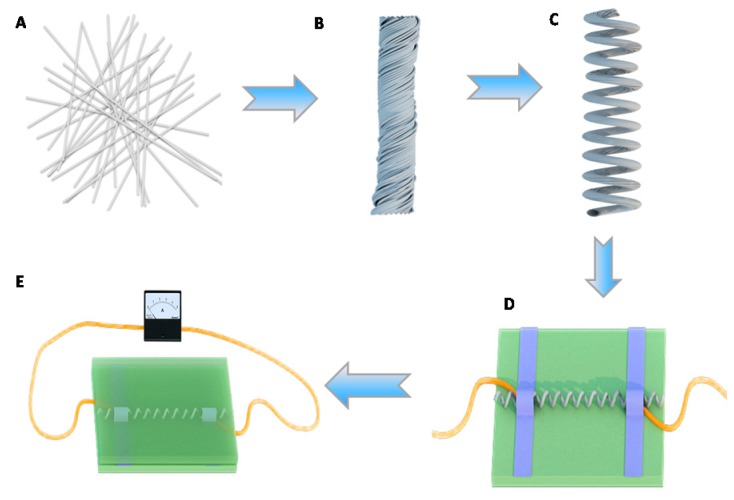
Schematic illustration of fabricating Ag nanowires/thermoplastic polyurethanes (AgNWs/TPU) strain sensor with a spring-like configuration. **A** Electrospinning of the TPU nanofibrous membrane and dip coating AgNWs. **B** The TPU/AgNWs strain sensor with twisted configuration. **C** The TPU/AgNWs strain sensor with spring-like configuration. **D** The attachment of two electrodes. **E** Detecting of human motion.

Figure 1 schematically illustrates the fabrication of an AgNWs/TPU sensor with a spring-like configuration through a controlled fabrication and a simple, scalable, and economic process.

### 2.1. Materials

TPU was purchased from Bayer (Germany). (Silver nitrate) AgNO_3_ was bought from Sinopharm Chemical Reagent Co., Ltd. (Shandong, China). Poly(vinylpyrrolidone) (PVP) was obtained from Usolf Chemical Technology Co., Ltd. (Shanghai, China). Acetone, and *N*,*N*-dimethylformamide (DMF) were purchased from Sinopharm Chemical Reagent Co., Ltd. (Shanghai, China). Ethylene glycol (EG) were supplied by Tianjin Fuyu Fine Chemical Co., Ltd. (Tianjin, China). All materials were used without further purification.

### 2.2. Electrospinning of TPU Nanofibrous Membrane (Schematic A)

Electrospinning is a versatile technique to fabricate nanofibers in the form of individual fibers, parallel fibers, or non-woven nanofibrous membrane. For the fabrication of TPU nanofibrous membrane, 1.3 g TPU was dissolved in 8.7 g DMF and acetone (1:1) sequentially at first, and the solution was heated to 40° for 2 h to make them completely dissolved and form a 13 wt % homogeneous precursor solution. Then the precursor solution was pumped to a plastic syringe with a stainless needle tip. The spinneret had a diameter of 0.26 mm that served as the positive electrode, to which a high voltage of 13 kV was supplied between the precursors solution and the collector by a power supply (DW-P303-1ACFO, Tianjin, China). In this case, a stainless steel roller enveloped with aluminum foil was employed as the collector with the speed of 240 rpm. The fluid flow rate of the precursor solution was controlled as 0.02 mL/min by a syringe pump. The distance between the needle tip and the collector was 12 cm. In the process of electrospinning, relative humidity and environmental temperature was 45% and room temperature, respectively.

### 2.3. Fabricating of TPU/AgNWs Nanomembrane (Schematic B)

AgNWs were synthesized by the reduction of the AgNO_3_ in EG with PVP as follows: 0.205 g AgNO_3_ added to 10 mL EG to form solution A, and 0.467 g PVP were added to 10 mL EG to form solution B. Then they were stirred for 1 h to form homogeneous solution separately. A total of 50 µL FeCl_3_ aqueous solution with a concentration of 0.1 mol/L was added to solution B. After that, solution A was added to solution B. Then the solution was heated at 160 °C in an oil bath without air. AgNWs were synthesized while the solution turned into a gray color. After that, the grey solution was centrifuged at 8000 rpm for 10 min. Lastly, the obtained AgNWs solution was washed with ethanol to remove the retained PVP and EG.

To obtain the TPU/AgNWs nanomembrane (20 × 20 cm^2^), the obtained AgNWs were dispersed in ethanol at first and the concentration was controlled to be 26.0 mg·mL^−1^. The electrospinning white, porous TPU membrane was immersed into the AgNWs solution for 10 min as a cycle. Taking advantage of the wicking effect, the silver nanowires were spontaneously absorbed into the gap of the nanofibrous fibers or coated on the surface of the TPU membrane at room temperature due to the principle of capillary action [33]. The color of the TPU membrane changed from white to gray. Thus, the AgNWs conductive networks were built over the TPU/AgNWs nanomembrane surface. The content of AgNWs in the TPU/AgNWs nanomembrane can be effectively adjusted by varying dip-coating cycles, which adjusts the conductivity. For the aim of increasing the conductive networks of silver nanowires, three dip-coating cycles were adopted [32]. The content of AgNWs ranged from 11.67% to 52.87% while the dip coating cycles increased from one to three. In addition, the conductivity increased sharply from 2.34 to 3990 S·cm^−1^ after cycles of three. After exchanging the impurities with deionized water and drying at room temperature, a TPU/AgNWs membrane was obtained.

### 2.4. The Sensor with Spring-Like Configuration (Schematic B–C)

Starting from a TPU/AgNWs membrane, spring-like configuration with uniform small loops could be formed by a rotating device [34] as follows. The TPU/AgNWs membrane was placed with one end fixed onto a motor, and the other end fixed onto an iron support. It should be noted that the iron support should be moveable so that it is feasible to meet the demand of the deformation of the membrane. With the rotating of the motor at a speed of 100 rpm, the membrane was rotated and turned into the twisted configuration gradually at first, as shown in schematic b. Additionally, the motor changed the rotating speed to 60 rpm, and the desired spring-like fiber was obtained in the end, as shown in schematic c. A total of 20 cm of fibers can produce 5 cm of a spring-like fiber.

### 2.5. Electrode Fabrication (Schematic D)

For the aim of manifesting the wearability of the sensor with spring-like configuration, two electrodes were fabricated by mounting two copper wires onto two ends of the sensor, and the distance between two electrodes was 10.0 mm. Additionally, to enhance the stability of the electrodes, copper conductive tapes were introduced to immobilize the electrodes.

### 2.6. Properties of Strain Sensor Measurements

The morphologies of the resultant sensor with spring-like configuration were observed by the scanning electron microscope (SEM, XL-30S FEG, Philips, Amsterdam, The Netherlands). The composition was characterized through energy-dispersive X-ray spectroscopy (EDS, Hitachi S4800, Tokyo, Japan). X-ray diffraction (XRD, Rigaku D/max-2400, Tokyo, Japan) patterns were used to observe the microstructure of the sensor in the 2θ range of 10°–80° at room temperature. Both the electrical characteristics were measured by an Agilient 9200 high resistance meter system (schematic e).

## 3. Results and Discussion

### 3.1. The EDS Spectrum of the Strain Sensor with Spring-Like Configuration

**Figure 2:**
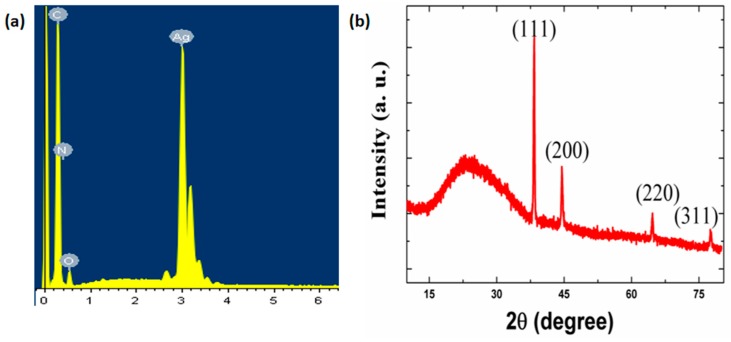
(**a**) The EDS spectrum of the strain sensor. (**b**) The XRD map of the strain sensor.

The point scanning of EDS is shown in Figure 2a, which demonstrates the elementary composition of the TPU/AgNWs sensor. On one hand, the EDS result presented that, in addition to the C, N, and O elements, the Ag element was also the main element constituting the sensor, which could be attributed to the existence of Ag nanowires. Additionally, the experimental atomic percentage of the Ag element calculated from the EDS spectrum is 52.87 wt %, which led to the high conductivity of the strain sensor. No other impurities were detected from the EDS spectrum.

### 3.2. The XRD Map of the Strain Sensor with Spring-Like Configuration

For the aim of confirming the structure of the sensor, the XRD map is shown in Figure 2b, which demonstrates the crystallographic characteristics of the sensor. Four typical characteristic diffraction peaks appeared at 38.32°, 44.47°, 64.59°, and 77.59° that could be ascribed to the (1 1 1), (2 0 0), (2 2 0) and (3 1 1) crystal diffraction planes of Ag nanowires, respectively [35,36]. Furthermore, there was a strong and broad peak at 20° corresponding to the amorphous phase of TPU [35].

### 3.3. The SEM Image of the Strain Sensor with Spring-Like Configuration

**Figure 3:**
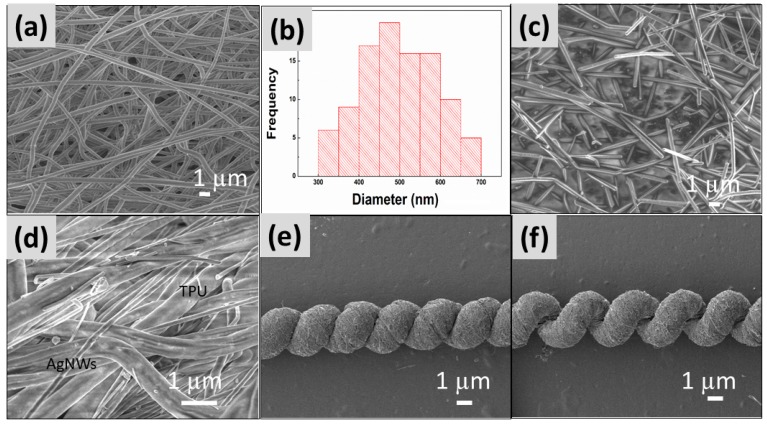
(**a**) SEM image of the electrospun TPU nanofibrous membrane. (**b**) Diameter distribution of an electrospun TPU membrane. (**c**) SEM image of AgNWs. (**d**) SEM image of TPU/AgNWs membrane. (**e**) SEM image of the strain sensor with spring-like configuration under a 0% strain. (**f**) SEM image of the strain sensor with a spring-like configuration under 50% strain.

The SEM image of electrospun TPU nanofibrous membrane was shown in Figure 3a, and it showed the average diameter was 486 nm. Figure 3b was the histogram of diameter distribution of the electrospun TPU membrane. Figure 3c presented the SEM image of AgNWs, which showed the average diameter and length of AgNWs were about 180 nm and 10 μm, respectively. The SEM image of the TPU/AgNWs strain sensor in a spring-like configuration was shown in Figure 3d, and the nanowires were found to be randomly distributed on the TPU surface or among the TPU nanofibers successfully.

The spring-like configuration was derived from the moderate fibrous membrane gradually. In the present case, the length and width of the initial film were 20.5 cm and 3.0 mm, respectively. In the process of fabricating the spring loops, the membrane was formed into the twisted fibers with 19 cm length and 220 µm width at first, and then the fibers of spring-like configuration with a loop of 200 µm in width. In particular, it should be noted that about eight loops could exist over a 1 cm range. Figure 3e shows the SEM image of the sensor with a spring-like configuration in its original state. The configuration could be considered as a miniaturized version of conventional springs from the appearance. The sensor could be straight over a long range, and the surface of the sensor was very smooth. Each loop of the springs was uniform and in a tight arrangement. Additionally, thanks to the spring-like configuration, a large proportion of AgNWs could be rolled into the hierarchically arranged fibers, which avoids AgNWs falling off from the sensor during the mechanical deformation. It should also be noted that each loop in the opening state of the springs in the stretching state was also uniform, and the sensor was straight, as illustrated in Figure 3f.

### 3.4. Stretchability Test

**Figure 4:**
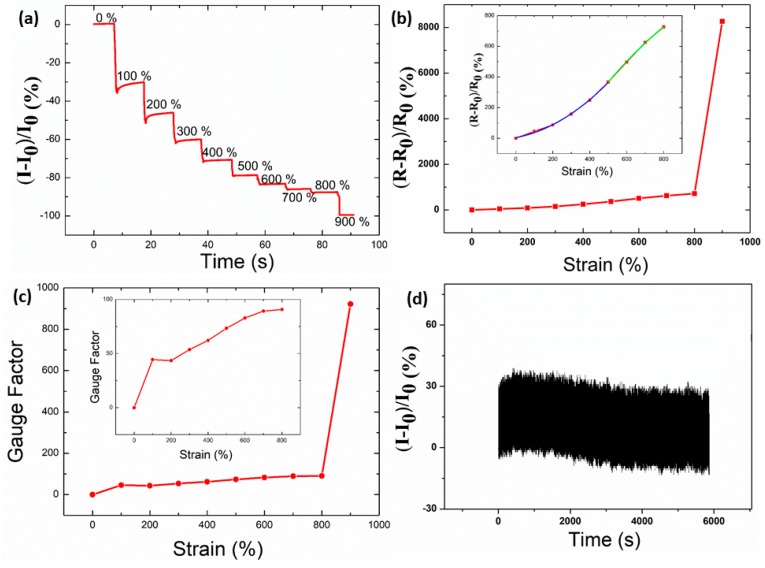
(**a**) Current responses of the sensor under different strains. (**b**) Typical relative resistance-strain curve of the strain sensor with a spring-like configuration. (**c**) The gauge factor-strain curve of the strain sensor with spring-like configuration. (**d**) Long-term stability test of a TPU/AgNWs strain sensor for 1000 stretching-releasing cycles from a 0% to 100% strain.

It is noted that AgNWs accepted as functional sensing elements have a large aspect ratio, so they can conduct with each other to make contact ideally. In this case, the conductivity was determined to be 3990 S·cm^−1^ based on the sensor in the twisted state. To detect the stretchability that the sensor could tolerate, it was stretched along the axis at 100% strain at each step. The relative variation in current of the strain sensor with a fixed voltage of 0.01 V under axial strain from 0% to 900% is shown in Figure 4a. It was interesting to detect that the limitation of the strain that the sensor could sustain was 900%, at which point the sensor ruptured. The maximum rupture strain of TPU/ AgNW fibers with 14.6 wt % AgNW by wet spinning was 786%, and it was nearly 150% with 50.1 wt % AgNW [37]. The polyurethane (PU)/AgNW fibers with wrinkled microstructures through writing Ag nanowires’s ink layer on pre-strained commercial PU fibers showed high stretchability of 400% [38]. AgNWs/Poly(3,4-ethylenedioxythiophene)-poly(styrenesulfonate) (PEDOT:PSS)/PU film encapsulated with PDMS could tolerate a strain up to 240% [39]. The strain sensor based on electrospun TPU nanofibrous membrane and in situ polymerization of polyaniline (PANI) could tolerate a strain up to 165% at the point the sensor ruptured [40], while the strain that the electrospun TPU/Poly(vinylidene fluoride(PVDF) composite membrane could tolerate was less than 100% [41]. Additionally, it was difficult for the stretchable sensors to retain excellent conductivity when facing severe deformation in cases such as stretching, bending, twisting, and folding [29,42]. In this scenario, taking advantage of the high ratio of AgNWs, at the state where the AgNWs’s conductive network of the sensor was nearly broken, the conductivity of the sensor was still 5.06 × 10^−2^ S·cm^−1^, which also retains high sensitivity.

Figure 4a presents that current decreased sharply under various strains, which indicates the unique sensitivity to strain. We believed two factors could be attributed to this. One is the deformation of the spring configuration, and the other is that TPU has intrinsically stretchable components. In the process of stretching, the sensor underwent two stages. The first stage was the spring-like configuration recovering back to its shape and electrical characteristics in the original state. In this stage, the sensor behaved like a spring when the strain was less than 100%. Nevertheless, unlike the traditional spring made of rigid metal materials, the materials in the present case were stretchable inherently, which results in high stretchability that can be recoverable. When the sensor could not recover back to its original spring shape, this was the second stage. However, the sensor could act like a variable resistance to sense the deformation of the sensor.

To characterize the performance of the TPU/AgNWs strain sensor, the relative resistance change versus tensile strain was shown in Figure 4b. The scatter is the experimental data, and the line is the theoretical data. In this case, relative resistance change is defined as (Δ*R*/*R*_0_) × 100%. *R*_0_ and *R* are the original resistance and the variation in resistance, respectively. It can be seen clearly that a relative resistance change increased with applied strain. However, it increased dramatically after 800% strain, and it may be the reason that the conductive network was broken while the strain sensor was ruptured, which leads to a rapid increase in the resistance. As for the region of 0% to 800% strain, the Δ*R*/*R*_0_ plotted against the applied strain could be divided into two distinct stages, as shown in the insert of Figure 4b. The Δ*R*/*R*_0_ increased slowly in the region of 0% to 500% strain, and this may be the reason that the AgNWs network was tighter thanks to the spring-like configuration, and connected the gaps between neighboring TPU fibers. However, when the applied strain was higher, the AgNWs conductive network became looser, and many conductive networks may be destroyed. Therefore, a sharp resistance increase could be observed. The curve was perfectly fitted quadratically in the 0%–800% strain range. The fitted equation was y = 4.5564 + 0.2206x + 9.9522x^2^ from 0% to 500% strain, and y = −532.8489 + 2.1657x + 0.0007387x^2^ from 500% to 800% strain. The coefficients of determination (*R*^2^) was determined to be 0.9977 and 0.9987, respectively. A gauge factor is of great importance to evaluate the superiority of the resulting sensor since it can represent the sensitivity of the sensor to a certain strain [27,43]. It is defined as (Δ*R*/*R*_0_)/(Δ*L*/*L*). In this case, L is the original length of the sensor. Figure 4c presents the gauge factor of the sensor under strain from 0% to 900%. In the present case, the gauge factor was 44.43 under 100% strain.

For obtaining the mechanical stability of the TPU/AgNWs strain sensor with spring-like configuration, a cyclic stretch–release test employing 100% strain was implemented. The stretch rate was controlled to be 20 mm/s, and the time was about 6 s for a cycle. The TPU/AgNWs strain sensor displayed good repeatability and stability up to 20,000 cycles under a repeated stretching 100% strain, and 1000 cycles was shown in Figure 4d. The current variation corresponded well to the value shown in Figure 4a under 100 strain. Additionally, it can be seen that the current variation in real-time recorded was very uniform, and no clear difference of the resistance change amplitude in the initial and final cycles was visible, which indicates the excellent durability of the TPU/AgNWs strain sensor for applications in our daily life.

### 3.5. Stability Test

**Figure 5:**
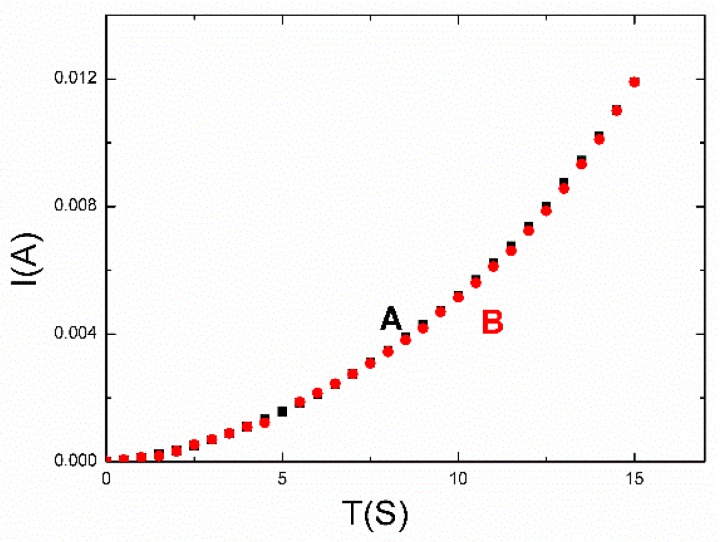
Stability tests of the sensor. The I–V characteristics of the sensor are shown at the origin state (curve A) and after being left for six months (curve B).

To detect the stability of the sensor, it was laid aside for six months at room temperature. Curve A and curve B in Figure 5 are the I–V characteristic curves of the strain sensor at the origin state and after being left for six months, respectively. Curve A and curve B are nearly coincident, which indicates no degradation in the conductivity. The strain sensor could maintain the original electrical characteristic due to the spring-like configuration that avoided the AgNWs falling off.

### 3.6. Sensitivity of the Bending Test

**Figure 6:**
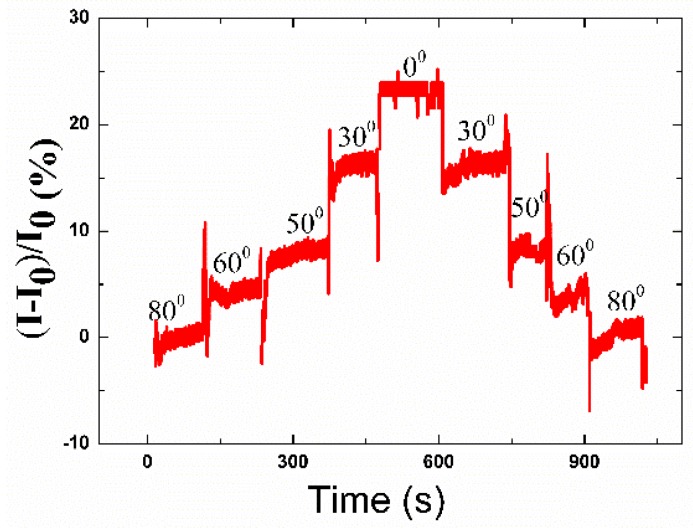
The currents of the strain sensor facing bending of various angles.

The sensitivity and recovery to shape change, such as bending, folding, and curlating, etc., is very important for practical application [44]. To simulate the motion of the bending, the sensor was placed on two stages with a distance of about 1.8 cm. The bending degree of the strain sensor could be adjusted by controlling the distance between two adjacent stages. For example, one side of the mechanical stage was moved to obtain the ideal bending angle of the sensor at 80°, 60°, 50°, 30°, and 0° step-by-step, and then 30°, 50°, 60°, and 80°, as shown in Figure 6. It should be noted that the strain sensor showed a sharp response speed facing different bending angles.

### 3.7. Sensitivity of Pulse Pressure Monitoring

**Figure 7:**
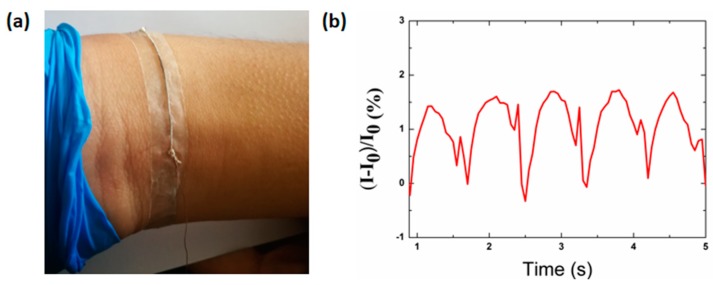
(**a**) Photograph of strain sensor attached on the wrist. (**b**) Relative current response of the strain sensor in monitoring the pulse pressure of a human wrist (physical condition-age: 40 years, height: 170 cm, and weight: 69.5 kg).

The successful application of the wearable sensor is associated with slight motion, such as the vasoconstriction and vasodilation of blood vessels, so as to recognize real-time pulse. In this case, the strain sensor was attached on the wrist (Figure 7a) to detect the radial artery pulse waveform, as shown in Figure 7b. Figure 7b shows the current signal recorded under normal conditions for 4.5 s. The amplitude and the frequency of wrist pulses can be obtained accurately in real time, for each peak represents one pulse cycle. By means of the frequency of wrist pulses, the heart rate can be determined to be 83 beats per minute under normal conditions.

### 3.8. Sensitivity of Finger Motion Monitoring

**Figure 8:**
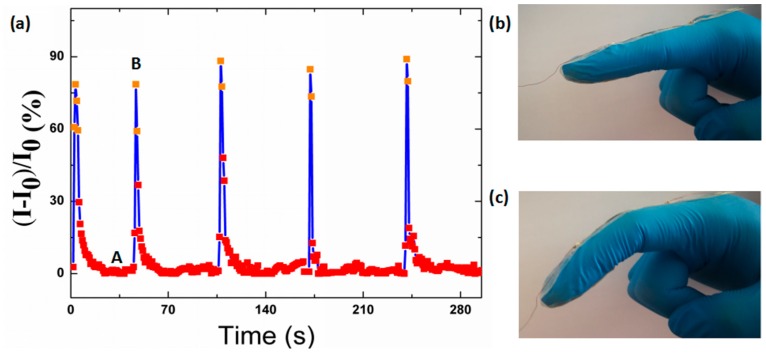
(**a**) Response of the strain sensor with spring-like configuration worked as wearable sensors in monitoring forefinger bending. A: The relaxing state of the strain sensor when the forefinger was bent suddenly. B: The maximum relative current change of a strain sensor to the response of bending the forefinger at the point the forefinger recovers to the original state. (**b**) Photographs of the forefinger in the original state. (**c**) Photographs of the forefinger in the bending state.

The motion of the finger, which is also regarded as one of the most sophisticated human actions, was also detected. In this scenario, the relative current response of the strain sensor to the instantaneous bending and unbending actions of the forefinger is shown in Figure 8. In a cyclical movement, point A is the relaxing state of the strain sensor in which the forefinger was bent suddenly, while the strain sensor showed sharp sensitivity to the motion. Point B is the maximum relative current change of a strain sensor to the bending motion, and it should be pointed out that the relative current changes at point B differ somewhat for each cycle because the degrees of the bending are not absolutely unanimous. Additionally, when the forefinger straightened suddenly, the current of the strain sensor recovered to the original electrical characteristic sharply. Additionally, the current remained stable when the finger was in the original state.

**Figure 9:**
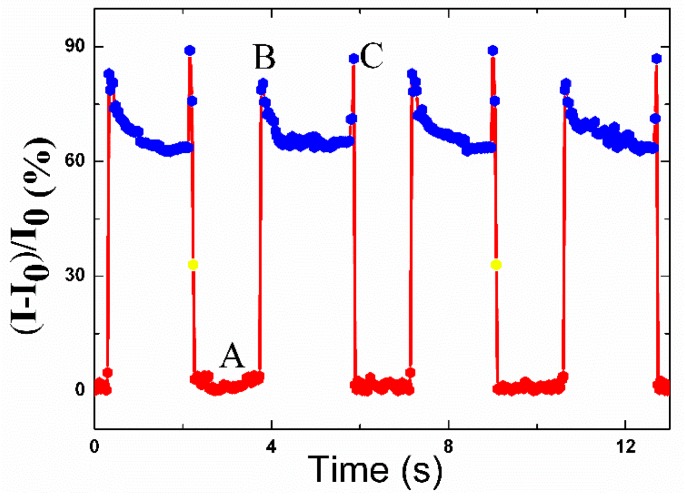
Response of the strain sensor with spring-like configuration worked as wearable sensors in monitoring the stability of forefinger bending. A: The relaxing state of the strain sensor at the point the forefinger was bent suddenly. B: The maximum relative current variation of the strain sensor to the response of bending the forefinger. C: The bending state of the strain sensor at the point the forefinger recovered to the relaxing state suddenly.

To demonstrate the stability of the strain sensor to the bending of the forefinger, the forefinger maintained the states of unbending and a fixed bending degree for a certain time in each cycle. In Figure 9, the current of the strain sensor varies simultaneously with the bending and then maintains stable while the forefinger bends to a certain angle, which demonstrates the fast response and high sensitivity.

**Figure 10:**
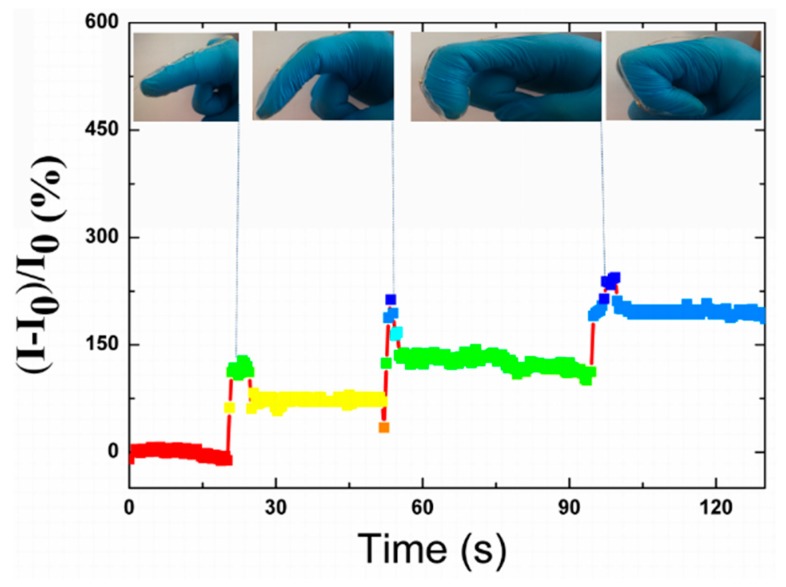
Relative current responses of a strain sensor in detecting various degrees of the forefinger’s bending. Inserts are the images of various bending degrees.

Thanks to the sensitivity of the strain sensor when it underwent various bending degrees, it was applied to monitor various bending degrees of the forefinger precisely (Figure 10). In this case, the strain sensor showed a sharp sensitivity to various degrees.

### 3.9. Sensitivity to Knee Motion

**Figure 11:**
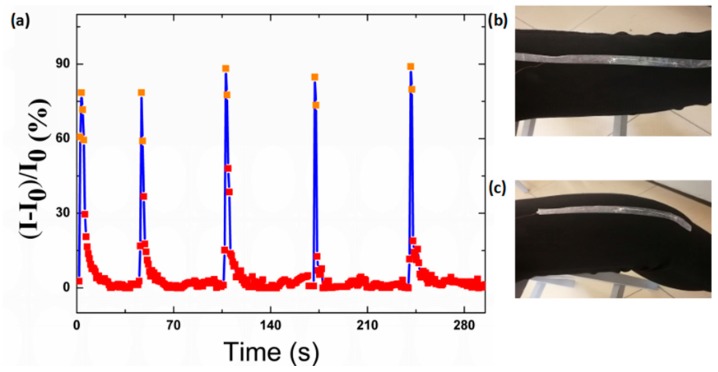
(**a**) Relative current change of the strain sensor in monitoring the bending of the knee. (**b**) Photograph of the knee in the original state. (**c**) Photograph of the knee in the bending state.

The sensitivity to the motion of the knees was also tested (Figure 11). The knee was in the standard state at first, and then it was bent suddenly. It can be seen that the sensor shows a unique sensitivity to the motion of the knee. Moreover, it could recover to the original electrical characteristic when the knee recovered to the original standard level state. It should also be noted that the electrical signal may differ somewhat in the standard level state for the physiological shake of the knee.

### 3.10. Pressure-Resolved Response

**Figure 12:**
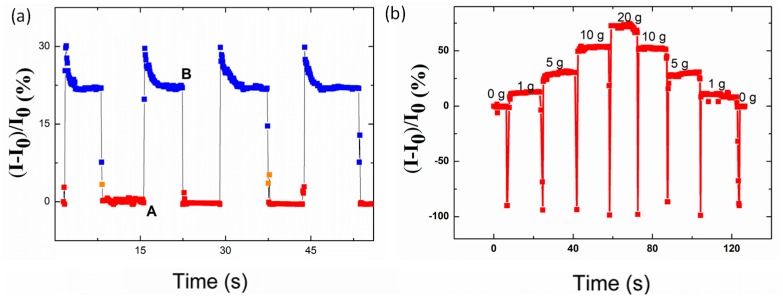
(**a**) Current response of the sensor to the tapping of the finger. A: The relaxing state of the strain sensor at the point the pressure was assessed by the index finger. B: Thetransition point from a pressure state to a relaxed state when a tapping was withdrawn suddenly. (**b**) Current response of the sensor to the various weights.

The pressure sensitivity is essential in the practical application [45]. For the purpose of acquiring a unique sensitivity, four spring-like configuration sensors (3 cm in length and 0.5 mm in width) were connected by a copper wire to form a parallel circuit to enlarge the area. The distance between the two nanofibers was 0.5 mm. Then, the sensor was attached to the arm by the 3M tape. In the present case, to simulate various conditions, the pressure was assessed by the index finger at first, and the pressure was maintained for about 8 s. For the reason that the pressure was not stable for the physiologic tremor of the finger, the curve was not smooth, as shown in Figure 12a. However, the recovery and repeatability were all high. Additionally, the sensitivity of a strain sensor to a pressure is defined as (Δ*R*/*R*_0_)/*p*, where *R*_0_ is the original resistance and Δ*R* is the current change when tapping is applied on the strain sensor. Thus, the sensitivity to the pressure applied by the tapping can be determined to be 3.64 k Pa^−1^.

Then, the pressure was assessed by the weight of various mass slightly. As shown in Figure 12b, the sensor showed unique sensitivity to the various mechanical pressure like a variable resistor. It can be seen that the sensor had a fast response time, and the current recovered to the initial value fully after withdrawing the tapping. Under mechanical tapping once more, the device repeated almost the same value of the previous circle.

## 4. Conclusions

In conclusion, a stretchable strain sensor with perfect spring loops arrangement over a long distance was demonstrated via a simple and cost-effective method. Thanks to the spring-like configuration, the sensor can tolerate a high strain up to 900%, and it could survive 10,000 cycles of 100% stretch–release testing. It presented a high conductivity up to 3990 S·cm^−1^ due to a high ratio of AgNWs. The TPU/AgNWs strain sensor could be applied for stretch, bending, and pressure sensing. As demonstrated, this TPU/AgNWs strain sensor could be used to detect major and minor human body motions, and showed remarkable sensitivities as stretchable sensors, which implied the great application of the new spring-like configuration in a wearable electronic device. The fabrication method presented in this study are projected to pave a new way to work in the field of healthcare human-machine interaction, sound recognition, artificial intelligence, electronic skin, and others.
